# Hyposulphite of Soda

**Published:** 1891-07

**Authors:** J. W. Marsh


### HYPOSULPHITE OF SODA.

By J. W. Marsh, D. V. S.

---

My attention was attracted to the article by E. N. Sheppard, in the March number of The Journal in regard to the use of Hyposulphite of Soda.

I find it a most valuable remedy in all my cases of Azoturia, since I have commenced to use it. Under the old line of treatment, in cases where my patient was prostrated and showed great restlessness, my prognosis was generally unfavorable. Since I have employed the Hyposulphite the greater number have made a recovery and I most heartily recommend its use in these cases.

